# Drug-Related Problems and Recommendations Made during Home Medicines Reviews for Sick Day Medication Management in Australia

**DOI:** 10.3390/medicina60050798

**Published:** 2024-05-11

**Authors:** Mimi Truong, Connie Van, Kamal Sud, Wubshet Tesfaye, Nerida Croker, Shrey Seth, Ronald Lynel Castelino

**Affiliations:** 1 School of Pharmacy, Faculty of Medicine and Health, The University of Sydney, Sydney 2006, Australia; 2 Nepean Kidney Research Centre, Department of Renal Medicine, Nepean Hospital, Nepean and Blue Mountains Local Health District, Kingswood 2747, Australia; 3 Sydney Medical School, Faculty of Medicine and Health, The University of Sydney, Sydney 2006, Australia; 4 School of Pharmacy, Faculty of Health and Behavioural Sciences, The University of Queensland, Brisbane 4072, Australia; 5 Meditrax, Drummoyne 2047, Australia; 6 Pharmacy Department, Blacktown Hospital, Western Sydney Local Health District, Blacktown 2148, Australia

**Keywords:** drug-related problems, chronic kidney disease, acute kidney injury, sick day management, medication therapy management, descriptive study

## Abstract

*Backgrounds and Objectives*: Using certain medications during an intercurrent illness can increase the risk of drug related problems (DRP) occurring such as acute kidney injury (AKI). Medications that increase this risk include sulfonylureas, angiotensin converting enzyme inhibitors, diuretics, metformin, angiotensin receptor blockers, non-steroidal anti-inflammatories drugs, and sodium glucose co-transporter 2 inhibitors (SADMANS). Sick day medication guidance (SDMG) recommends withholding SADMANS medications during an intercurrent illness where adequate fluid intake cannot be maintained. But uptake of these recommendations is poor, and it is not known whether Australian pharmacists currently provide these recommendations during home medicine reviews (HMR) as per SDMG. We aimed to gain an understanding of the characteristics of DRP identified by pharmacists during HMR, especially those relating to SADMANS medications. *Materials and Methods*: We conducted a retrospective audit of 201 randomly selected HMR reports, conducted by accredited pharmacists from 2020 to 2022, that were analysed in 2023. All DRP and recommendations were categorised using a modified DOCUMENT system. *Results*: Overall, over 98% of participants experienced a DRP and a total of 710 DRP were found, where participants experienced an average of 4.0 ± 2.0 DRP each. Non-SADMANS medications accounted for 83.1% of all DRPs, with nervous system medications contributing the most. Common problems seen in non-SADMANS medications were related to toxicity, over/underdosing and undertreating. Diuretics contributed most to DRP in SADMANS medications. Problems with SADMANS were mainly related to toxicity and contraindications. No pharmacists provided SDMG despite 71.1% of participants using at least one SADMANS medication. *Conclusions*: We conclude that DRP remain prevalent in community pharmacy settings. Sick day recommendations were not provided in the HMRs included in our study, possibly due to lack of pharmacist knowledge and awareness. To ensure best practice, more research should be conducted to determine pharmacists’ knowledge of and barriers to provision of sick day recommendations.

## 1. Introduction

Drug related problems (DRP) are highly prevalent in community-dwelling older people (>65 years) and have contributed to 2.5% of all hospitalisations in Australia, with an estimated cost of AUD 1.4 billion annually [1]. This is comparable with countries with similar demographics including Canada (CAD 35.7 million) [2] and the United States (USD 177.4 billion) [3]. People who have chronic kidney disease (CKD) experience multiple factors which increase their risk of experiencing DRP—such as taking multiple medications [4,5]. On average, patients with CKD take 10 to 13 medications [6], which increases the risk of DRP like adverse drug reactions and drug-drug interactions [4,5]. It has also been observed that CKD patients managed on haemodialysis experience more DRP than those who do not require haemodialysis [6]. Since CKD alters physiology, the pharmacokinetic and pharmacodynamic properties of several medications is also altered leading to increased risk of DRP in comparison to those without CKD [7,8,9,10]. Other DRP that burden CKD patients include ineffective treatment, dosing problems and inappropriate prescribing, with causative medications including antibiotics and oral anti-diabetic agents [9,11]. Inappropriately prescribed medications (defined as contraindicated/higher than recommended doses as per kidney function) in people with CKD ranges between 9.4% and 81.1% in community settings [11]. Furthermore, people with CKD who experience intercurrent volume depleting acute illness (such as vomiting and diarrhoea), are at an increased risk of experiencing adverse drug reactions such as acute kidney injury (AKI), hypoglycaemia, ketoacidosis, and hypotension [12,13,14]. AKI worsens CKD progression and increases the risk of morbidity and mortality [10,15,16].

To minimise the risk of these drug related problems, organisations from different countries including Kidney Health Australia, Diabetes Canada, and National Health Service’s Think Kidneys have recommended withholding certain medications during periods of acute illness [7,17,18]. These medications include sulfonylureas, angiotensin converting enzyme inhibitors (ACEis), diuretics, metformin, angiotensin receptor blockers (ARBs), non-steroidal anti-inflammatories drugs (NSAIDs), and sodium glucose co-transporter 2 (SGLT2) inhibitors, collectively referred to as SADMANS [19]. The SADMANS mnemonic was developed to remind health care professionals to advise patients to withhold these medications when they are acutely unwell.

It has been shown that pharmacists play a vital role in CKD management in community settings as they can apply expert medication knowledge to detect clinically relevant DRP across community and aged care settings [6,20]. In Australia, accredited pharmacists can identify and resolve actual or potential DRP during home medicines reviews (HMR), which are a government-funded service [21]. A review showed that HMR improve patient and prescriber understanding of medications, and that collaboration between pharmacists and prescribers can lead to 85% of DRP being resolved [22]. Kidney Health Australia have recommended that pharmacists discuss sick day action plans during this HMR service [17]. However, uptake of sick day medication guidance (SDMG) remains poor, with less than 15% of patients given SDMG by health care professionals (HCP) and only 5% acting on this advice [19]. Furthermore, a recent study found that >70% of patients hospitalised at a tertiary hospital in Australia with AKI were taking at least one SADMANS medication and 40% of these patients had hypovolaemia, vomiting and/or diarrhoea at admission [23,24].

Therefore, the main aim of this study was to gain an understanding of DRP currently identified during HMR and the recommendations made by pharmacists during the HMR service.

The specific objectives were as follows:

(1) Describe the characteristics of DRP identified by pharmacists, especially those relating to SADMANS medications and inappropriate use of medications as per kidney function.
(2) Describe recommendations made by pharmacists to general practitioners, including any recommendations to withhold medications during an acute illness.

## 2. Materials and Methods

### 2.1. Sampling, Study Population, and Ethics

A retrospective analysis of 201 HMR was performed, pertaining to people living in Australia, collected from one of Australia’s leading medication management review providers. The HMR were conducted between December 2020 to October of 2022 and each review represented a single participant as no repeat reviews were conducted. Ethics approval was granted by the Institutional Human Research Ethics Committee prior to commencement (2022/584).

### 2.2. Data Extraction, Coding, and Exclusion Criteria

Participant demographic information including age, sex, postcode, medical conditions, and medication history were collected from the HMR reports. The participant’s medical conditions were classified using the World Health Organisation’s *International Classification of Diseases* [25]. Comorbidities that increased the risk of AKI, including hypertension, diabetes mellitus (or type 2 diabetes), cardiovascular disease, and CKD were also recorded. CKD was classified according to the KDIGO staging: G1 (normal or high, eGFR > 90 mL/min/1.73 m^2^); G2 (mildly decreased, eGFR 60–89 mL/min/1.73 m^2^); G3a (mildly to moderately decreased, eGFR 45–59 mL/min/1.73 m^2^); G3b (moderately to severely decreased, eGFR 30–44 mL/min/1.73 m^2^); G4 (severely decreased, eGFR 15–29 mL/min/1.73 m^2^); and G5 (kidney failure, eGFR < 15 mL/min/1.73 m^2^) [26]. The Charlson comorbidity index [27] was calculated, where participants were considered to have a solid tumour if they were taking an antineoplastic agent and did not have a diagnosis of lymphoma or leukaemia at time of review. The participants’ medications were classified by World Health Organisation’s Anatomical Therapeutic Chemical and Defined Daily Dose (ATC/DDD) index 2023, which excludes complementary, homeopathic, and traditional medicinal products [28]. Medications were categorised according to if they were regularly taken or pro re nata (or ‘when required’) medications.

All DRP and pharmacist recommendations were divided into two categories: non-SADMANS and SADMANS medications, and were classified using a modified DOCUMENT system, a tool used in community pharmacy to report actual/potential DRP and clinical interventions [29,30]. Although the original DOCUMENT system was validated, modifications were necessary as it did not adequately capture all problems and recommendations made by pharmacists. Modifications to the DOCUMENT DRP classification system included addition of (T4) cautioning against toxicity and (NC) nonclinical (Table S1). Other modifications to DOCUMENT recommendations made by pharmacists included (R3a) drug change: cease, (R3b) drug change: initiate, (R3c) drug change: cease and initiate, (R8a) drug change: combination formulation, (R9a) review prescribed medicine, (R16a) information to nursing staff, (R20) nonclinical, (R0) not classifiable (Table S2).

Where pharmacists provided multiple recommendations for a single DRP, the overall recommendation was coded. For instance, the following recommendation “Consider assessing her blood pressure readings to ascertain if reduction of ramipril dose further to 2.5 mg daily is indicated” was classified as “dose decrease” rather than “monitoring: non-laboratory test”. All coding was completed by one researcher (M.T.) and cross-checked with another researcher (R.L.C.). Inappropriate use of medications in people with reduced kidney function was also recorded and defined as the use of medications in higher than recommended doses or contraindicated medication, as per previous studies [11,31].

**Figure 1:**
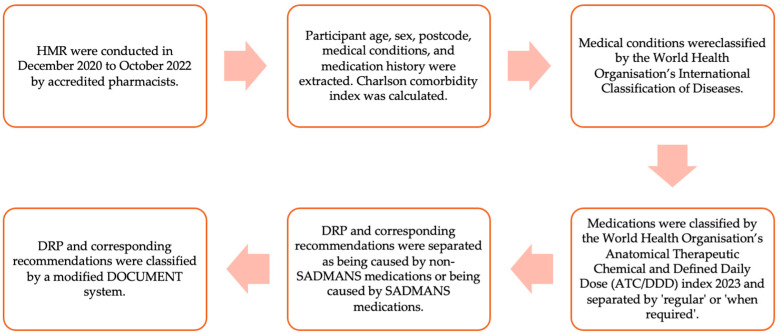
Summary of data extraction and coding methods.

HMR reports were excluded from the study if they were duplicate reports, or if participants were not taking medications at the time of review. A summary of data extraction and coding methods can be found in Figure 1.

### 2.3. Data Handing

Data analysis was performed using Microsoft Excel Version 16.84 and RStudio Version 16.84. All demographic data with normal distribution were presented as means (±SD) and as median (interquartile range) if not normally distributed. Descriptive statistics were presented as frequencies or proportions where appropriate.

## 3. Results

A total of 201 HMR reports were collected, and all were included for analysis as none met the exclusion criteria. The participants from this study were mostly male (52.2%) with a median age of 69 years and mainly resided in metropolitan areas of Australia (Table 1). The mean (±SD) number of medical conditions was 7.4 (3.0), and participants were taking, on average, 10.7 (4.0) regular medications and 2.2 (1.7) ‘when required’ medications (Table 1).

### 3.1. Non-SADMANS DRP and Recommendations Made by Pharmacists

DRP were highly prevalent and found in 98.5% (*n* = 198) of the participants, with an average of 4.0 ± 2.0 DRP overall. In total, 710 DRP were identified by the pharmacists and 83.1% (*n* = 590) of them related to non-SADMANS medications. The most common problems reported in non-SADMANS medications were cautioning against toxicity (16.6%, *n* = 98), condition undertreated (11.7%, *n* = 69), and other dose-related problems (7.6%, *n* = 45) (Table 2).

The most commonly used medications that caused these DRP pertained to medications used to treat the nervous system (incidence 28.0%, *n* = 215/448), alimentary tract and metabolism (24.4%, *n* = 161/667), and cardiovascular system (28.5%, *n* = 85/298) (Table 2). Recommendations most often made by pharmacists included laboratory test monitoring (8.3%, *n* = 59/710), drug change: initiate (3.8%, *n* = 27/710), dose decrease (3.1%, *n* = 22/710) (Table 2). Pharmacists also provided recommendations related to untreated conditions, which often included initiating nervous system medications, for instance addition of buprenorphine for unmanaged pain.

Reduced kidney function (eGFR < 90) was seen in 25.4% (*n* = 51) of participants; 14.4% (*n* = 29) of participants had reduced kidney function (without documented diagnosis of CKD), and 10.9% (*n* = 22) had CKD documented as a diagnosis. Of those with CKD, 9.1% (*n* = 2) were in stage G3a, 50.0% (*n* = 11) in G3b, 18.2% (*n* = 4) in G4, and none in G5. Also, 22.7% (*n* = 5) had CKD, but the stage and kidney function was not specified. Many participants also had risk factors for CKD, 45.3% (*n* = 91) had hypertension and 26.9% (*n* = 54) had diabetes and 17.4% (*n* = 35) had both. Only one patient had a recorded history of an AKI.

Of the 51 participants who had reduced kidney function, 86.3% (*n* = 44) were taking a medication that potentially required dose adjustments and/or were contraindicated (according to the *Australian Medicines Handbook* or product information) and 59.1% (*n* = 26) of these people experienced a DRP with these medications. A total of 36 DRP were found, with 41.7% (*n* = 15) caused by non-SADMANS medications (Table 3). DRP were found related to contraindications, inappropriate dosing, and issues around toxicity. There were a variety of inappropriately prescribed non-SADMANS medications, but the most common were anticoagulants (8.3%, *n* = 3), DPP-4 inhibitors (5.6%, *n* = 2), and statins (5.6%, *n* = 2) (Table 3).

### 3.2. SADMANS Related DRP, Recommendations, and SDMG

SADMANS medications were commonly used; 71.1% (*n* = 143) of participants used at least one SADMANS medication. Of those taking SADMANS medications, 43.4% (*n* = 62) took one, 28.0% (*n* = 40) took two, and 28.7% (*n* = 41) took more than three, with the most being five SADMANS medications. A total of 120 SADMANS DRP were identified by pharmacists, most associated with diuretics (28.3%, *n* = 34), metformin (25.8%, *n* = 31), NSAIDs (20.8%, *n* = 25), ARBs (14.2%, *n* = 17), ACEis (5.8%, *n* = 7), sulfonylureas (4.2%, *n* = 5), and SGLT2 inhibitors (0.8%, *n* = 1) (Table 4). Common problems seen in SADMANS medications were cautioning against toxicity (23.3%, *n* = 28), e.g., “The use of frusemide can be associated with potassium loss whilst spironolactone can be associated with potassium retention”. Other problems included toxicity caused by dose (13.3%, *n* = 16) and no indication apparent (8.3%, *n* = 10) (Table 4). The most common recommendations made by pharmacists included laboratory monitoring (*n* = 28), drug change: cease (*n* = 10) and drug change: cease and initiate (*n* = 7).

Only one episode of AKI was reported in a HMR that was conducted immediately post hospitalisation. The participant was taking the following SADMANS medications: candesartan (ARB), celecoxib (NSAID), hydrochlorothiazide (diuretic), and spironolactone (aldosterone antagonist and weak diuretic), which were all ceased following the AKI. The report did not mention whether the participant was acutely unwell prior to the AKI. No HMR warned of the risk of AKI or further dehydration occurring during acute illness due to SADMANS medications, nor did they provide any SDMG.

As previously mentioned, inappropriate prescribing was observed in people with reduced kidney function, in which SADMANS medications accounted for 58.3% (*n* = 21) of inappropriate prescribing cases in this group (Table 5). The most inappropriately prescribed SADMANS medication for people with reduced kidney function was metformin (*n* = 8), ARBs (*n* = 6) and diuretics (*n* = 3) (Table 5).

## 4. Discussion

Pharmacist-conducted HMR are an Australian Government funded strategy to ensure medication safety, aligned with the Australian Commission on Safety and Quality in Health Care’s Medication Safety Standard. This study provided an update on the number and nature of DRP seen in community settings and showed that DRP continue to be a significant issue in community settings, given that over 98% of participants, primarily older adults, in this study experienced at least one DRP. This audit also provided insight into lack of SDMG for people with CKD in the community during pharmacist-conducted HMR.

Like previous studies [9,23], ours showed that medication use was high in community-dwelling participants, with an average of ten regular medications and two ‘when required’ medications. Hyperpolypharmacy (10 or more medications) have been reported among community dwelling older people and occurs due to multimorbidity linked with aging [4,32]. Many adverse clinical outcomes are associated with polypharmacy, such as adverse drug reactions, drug interactions, cognitive impairment, and increased risk of falls [33]—which contributes to prefrailty/frailty, found in 8 to 16% of older people [4]. Our study found that on average, community-dwelling participants experienced 4.0 ± 2.0 DRP, which was consistent with previous studies that have reported an average of 2.7 to 3.9 DRP [34]. This reinforced the need for HMR, as HMR have decreased the number of DRP [21], improved quality of life [35], decreased the drug burden index [36], reduced hospitalisation [35], improved quality use of medicines [21], and improved parameters including blood pressure and glucose levels [35].

Our study found that DRP occurred in over 98% of participants who received an HMR. This is similar to findings from studies conducted by Nishtala et al. [36] and Truong et al. [30], who reported at least one DRP in 97% to 98% of people living in residential aged care facilities (RACF). It has been found that community-dwelling adults experience more DRP than those living in RACF, with one study reporting 4.8 ± 2.3 compared to 3.9 ± 2.0 DRP, respectively [37]. This was expected given that there is a greater focus on deprescribing in RACF to minimise polypharmacy [32], especially as goals of treatment shift toward better symptom management and improved quality of life, rather than to improve long term clinical outcomes [38]. Also, in comparison to those living in RACF, community-dwelling adults use more medications but have less opportunities for monitoring and intervention by health care professionals since medications are self-administered [37].

Overall, the three most common DRP found in this study were cautioning against toxicity, untreated conditions, and dose-related problems (including over/underdosing), which aligns with the findings of previous studies [6,9,22]. Since “cautioning against toxicity” was one of the most common problems found across all medication groups, this study indicated that pharmacists utilised HMR to identify both actual and potential DRP [36]. To address this DRP, pharmacists from our study typically recommended laboratory monitoring for potential toxicity, which has previously been shown to be the most implemented pharmacist recommendation [39], and the cause of many DRP [40]. Nervous system medications (including sedatives and hypnotics) were most cautioned against, which was not surprising given the risk of falls and other adverse outcomes associated with long term use of sedatives [5,41]. Similar to other studies [37], pharmacists also consistently exercised caution when nervous system conditions were undertreated and referred participants to other health care professionals rather than recommend medications, as compared to other body system conditions. This could be due to pharmacists taking a holistic approach to pain management [42]. For other body system conditions that pharmacists identified were untreated/undertreated, pharmacists were more likely to make recommendations to initiate medications, e.g., initiate pantoprazole for undertreated reflux [43,44,45]. Previous studies have reported issues with non-adherence as being one of the most common DRP found during HMR [22]. But in our study, adherence issues were only reported in the case of respiratory medications. It is not clear from other studies which medications had compliance issues [22]. Issues with lack of compliance for respiratory medications were likely due to lack of patient knowledge on the disease, poor understanding of the purpose of medications, and issues with the inhaler technique due to dexterity issues seen in older people [46]. Therefore, most pharmacists resolved these issues through patient counselling and education, which has been shown to be an important aspect of adherence. But further reinforcement is required from community pharmacists to maintain this adherence [46,47]. Other medications which caused DRP include alimentary tract and metabolism and cardiovascular medications, which has also been reported in other studies [37,48]. Hence, pharmacists should continue to pay close attention to these medication groups when monitoring for potential DRP.

SADMANS medications are important evidence-based medications indicated for many common conditions including type 2 diabetes, CKD, hypertension, and heart failure. These conditions were prevalent in this cohort, hence over 70% of participants used at least one SADMANS medication. The incidence of DRP for SADMANS medications was higher than non-SADMANS medications, with two of the most problematic SADMANS medications found to be diuretics and metformin. DRP caused by these medications have been reported in other studies [11,37] and occur as these medications are often inappropriately prescribed in people with CKD [48]. In our study, metformin was shown to be often inappropriately prescribed in patients with CKD due to renal insufficiency. This was expected from our study given that kidney function declines with age [31] and the median age of our sample was 69 years. Issues with metformin prescribing transpires across many countries, with one reason being lack of kidney function monitoring during treatment [9]. The issue was also seen in our study, since inappropriate prescribing of renally cleared or contraindicated medications in people with reduced kidney function was prevalent, with over 59% of these people experiencing a DRP relating to contraindications or inappropriate doses. This is similar to findings from a systematic review which reported inappropriate prescribing ranging between 25.9% to 52.6% in community dwelling Australian people, which is comparable with other countries including Canada and the United States [11]. However, reports of inappropriate prescribing were more prevalent in (above 70%) in other countries including Turkey, France, India, and Palestine [11].

In addition to contraindications/inappropriate prescribing, having no indication for using a medication was also often a problem, more so in SADMANS than non-SADMANS medications. Not having an indication mostly concerned diuretics, where pharmacists could not locate appropriate indications including heart failure diagnoses or where participants had no visible oedema. The authors determined that this could be due to incomplete medical history provided to the pharmacist by the referring doctor.

However, there were fewer issues of undertreated conditions or preventative therapy required for SADMANS medications in comparison to non-SADMANS medications. Less issues with conditions being undertreated may not have been observed in SADMANS medications because pharmacists may have lacked laboratory/test results to ascertain if blood glucose and blood pressure was adequately managed. In contrast, undertreated conditions typically referred to nervous system medications which pharmacists could more easily determine were inadequately controlled—for example, participants self-reporting unmanaged neuropathic pain requiring additional treatment. Further, fewer SADMANS medications require preventative prescribing to minimise side effects, which is likely why this type of DRP was not observed as often in SADMANS medications compared to non-SADMANS medications.

HMR is recommended as an important strategy for implementing SDMG by Kidney Health Australia in people with CKD [17]. However, as demonstrated by our study, this is not currently occurring in practice, highlighting an important research to practice gap [47]. Potential reasons for lack of SDMG provision may be due to pharmacists not being aware of at-risk patients or lack of knowledge and confidence on SDMG, as the recommendations are based on expert opinion rather than evidence from primary research studies [37,49]. This was highlighted in a recent scoping review where less than 15% of patients taking certain SADMANS medications were advised by health care professionals on how to adjust their medications during sick days [19]. Lack of sick day guidance from HCP may explain why a recent study found SADMANS medications present in approximately 70% of patients who had developed a community-acquired AKI, with 40% of them with symptoms of hypovolemia or dehydration due to vomiting or diarrhoea [23]. Additionally, patients taking SADMANS medications were more likely to have a mild AKI than those that were not [23]. As highly accessible HCP, pharmacists are in an excellent position to provide SDMG and reduce potential adverse outcomes associated with the use of SADMANS medications during an acute illness.

There are several limitations to our study. For one, all HMR were conducted by trained pharmacists from a single HMR service provider. Therefore their findings and recommendations may not be generalisable and representative of all reviews being performed across Australia. Another limitation is that there might have been an underestimation of the number of DRP if they were resolved prior to the HMR, or without the prescriber’s involvement and were not documented. However, given that this was a retrospective study, pharmacist behaviours and practices when conducting HMR were not influenced by the study, thus removing a source of bias. Lastly, no HMR included general practitioner responses, so we were unable to ascertain the appropriateness of pharmacist recommendations, although previous studies have shown acceptance rate of pharmacist recommendations ranging between 45 and 84% [34].

## 5. Conclusions

This study showed HMR remain a valuable tool for detecting DRP and that DRP remain highly prevalent in community settings. In our study, pharmacists did not provide any sick day recommendations for SADMANS medications for community-dwelling people despite the risks associated with using SADMANS medications during acute illness. This presents an opportunity to improve this area of care, hence further research is required to identify the knowledge gaps that pharmacists have, which may have prevented them from providing such recommendations.

## Figures and Tables

**Table 1:** Characteristics of study sample.

Demographic Information (*n* = 201)	Value
Median age (years) (IQR)	69 (24)
Sex (%)	
Female	96 (47.8%)
Male	105 (52.2%)
Remoteness (%)	
Major cities	147 (73.1%)
Regional	54 (26.9%)
Mean (±SD) number of medical conditions	7.4 ± 3.0
Top five medical conditions (*n* = 1482)	
Diseases of the circulatory system	245 (16.5%)
2. Mental, behavioural, or neurodevelopmental	214 (14.4%)
3. Endocrine, nutritional, or metabolic disease	202 (13.6%)
4. Diseases of the musculoskeletal system	181 (12.2%)
5. Diseases of the digestive system	125 (8.4%)
Mean (±SD) number of regular medications	10.7 ± 4.0
Top five regular medications used (*n* = 2155)	
Alimentary tract and metabolism	735 (34.1%)
2. Nervous system	448 (20.8%)
3. Cardiovascular	445 (20.6%)
4. Blood and blood forming organs	107 (5.0%)
5. Respiratory system	86 (4.0%)
Mean (±SD) number of ‘when required’ medications	2.2 ± 1.7
Top five ’when required’ medications used (*n* = 434)	
Nervous system	210 (48.4%)
2. Alimentary tract and metabolism	84 (19.4%)
3. Respiratory system	54 (12.4%)
4. Dermatological	37 (8.5%)
5. Musculoskeletal system	19 (4.4%)
Mean (±SD) Charlson comorbidity index	3.1 ± 2.1

**Table 2:** Frequent DRP and pharmacist recommendations relating to non-SADMANS medications.

Top Five Medications Associated with DRP	Incidence of DRP with Use	Four Most Frequent DRP Found with Use	Most Frequent Recommendation(s) to DRP
Nervous system	28.0% (215/448)	Cautioning against toxicity (61)	Monitoring: laboratory test (33)
		Condition undertreated (45)	Other referral required (13)
		Preventative therapy required (19)	Drug change: initiate (19)
		Other dose problem (16)	Dose decrease (5)
Alimentary tract and metabolism	24.4% (161/667)	Cautioning against toxicity (25)	Monitoring: laboratory test (15)
		No indication apparent (20)	Dose decrease (9)
		Condition undertreated (19)	Drug change: initiate (5)
		Other dose problem (17)	Dose decrease (11)
Cardiovascular	28.5% (85/298)	Toxicity caused by dose (24)	Dose decrease (5) Drug change: cease and initiate (5) Review prescribed medicine (5)
		Toxicity evident (12)	Dose frequency/schedule change (7)
		Other dose problem (10)	Dose frequency/schedule change (7)
		Contraindications apparent (9)	Dose decrease (3)
Blood and blood forming organs	45.7% (32/70)	Cautioning against toxicity (12)	Monitoring: laboratory test (8)
		Toxicity caused by dose (5)	Review prescribed medicine (2)
		Prescribed dose too high (2)	Dose decrease (1) Monitoring: laboratory test (1)
		Contraindications apparent (2)	Monitoring: laboratory test (2)
Respiratory	29.1% (25/86)	Taking too little (7)	Education/counselling session (2) Drug change: cease and initiate (2) Refer to prescriber (2)
		Condition undertreated (5)	Drug change: initiate (3)
		Erratic use of medication (2)	Education/counselling session (2)
		Other dose problem (2)	Drug change: cease (1) Refer to prescriber (1)

**Table 3:** Inappropriately prescribed non-SADMANS medications in people with reduced kidney function.

Medication or Drug Class	How the Medication Was Inappropriately Prescribed	Pharmacist Recommendation
Anticoagulants (3)	Prescribed dose too high (1)	Dose decrease (1)
	Prescribed dose too low (1)	Dose increase (1)
	Contraindications apparent (1)	Refer to prescriber (1)
DPP-4 inhibitors (2)	Prescribed dose too high (1)	Dose decrease (1)
	Contraindications apparent (1)	Drug change: cease and initiate (1)
Statins (2)	Cautioning against toxicity (2)	Drug change: cease and initiate (1) Dose decrease (1)
Atenolol (1)	Contraindications apparent (1)	Drug change: cease and initiate (1)
Cyclosporin (1)	Cautioning against toxicity (1)	Monitoring: laboratory test (1)
Digoxin (1)	Cautioning against toxicity (1)	Monitoring: laboratory test (1)
Fenofibrate (1)	Prescribed dose too high (2)	Dose decrease (2)
Hydroxychloroquine (1)	Toxicity caused by dose (1)	Monitoring: laboratory test (1)
Hydroxycarbamide (1)	Contraindications apparent (1)	Monitoring: laboratory test (1)
Nitrofurantoin (1)	Toxicity caused by dose (1)	Drug change: cease (1)
Sucralfate (1)	Contraindications apparent (1)	Refer to prescriber (1)

**Table 4:** Frequent DRP and pharmacist recommendations relating to SADMANS medications.

SADMANS Medication	Incidence of DRP with Use	Four Most Frequent DRP Found with Use	Most Frequent Recommendation(s) to DRP
Sulfonylureas	38.5% (5/13)	Cautioning against toxicity (2)	Monitoring: laboratory test (2)
		Laboratory monitoring (2)	Monitoring: non-laboratory test (1)
		Toxicity caused by dose (1)	Dose decrease (1)
ACEis	17.5% (7/40)	Toxicity caused by dose (4)	Dose decrease (1) Monitoring: non-laboratory test (1) Drug change: cease (1) Drug change: cease and initiate (1)
		Contraindications apparent (1)	Drug change: cease and initiate (1)
		Cautioning against toxicity (1)	Drug change: cease and initiate (I)
		Condition undertreated (1)	Dose increase (1)
Diuretics	69.4% (34/49)	Cautioning against toxicity (9)	Monitoring: laboratory test (7)
		No indication apparent (6)	Drug change: cease (3)
		Toxicity caused by dose (5)	Monitoring: laboratory test (3)
		Condition undertreated (5)	Refer to prescriber (3)
Metformin	70.5% (31/44)	Laboratory monitoring (6)	Monitoring: laboratory test (5)
		Cautioning against toxicity (5)	Monitoring: laboratory test (4)
		Prescribed dose too high (4)	Dose decrease (3)
		Contraindications apparent (3)	Dose decrease (1) Drug change: cease (1) Drug change: cease and initiate (1)
		Toxicity caused by dose (3)	Monitoring: laboratory test (1) Drug change: cease (1) Drug change: cease and initiate (1)
ARBs	29.3% (17/58)	Cautioning against toxicity (5)	Monitoring: laboratory test (3)
		Toxicity caused by dose (3)	Monitoring: non-laboratory test (2)
		Contraindications apparent (2)	Monitoring: laboratory test (2)
		Condition untreated (2)	Dose frequency/schedule change (1) Monitoring: non-laboratory test (1)
NSAIDS	39.7% (25/63)	Cautioning against toxicity (6)	Drug change: cease and initiate (2)
		No indication apparent (4)	Drug change: cease (3)
		Preventative therapy required (4)	Drug change: initiate (3)
		Toxicity evident (3)	Monitoring: laboratory test (1) Drug change: cease and initiate (1) Refer to prescriber (1)
SGLT2 inhibitors	9.1% (1/11)	Contraindications apparent (1)	Drug change: cease (1)

**Table 5:** Inappropriately prescribed SADMANS medications in people with reduced kidney function.

Medication or Drug Class	How the Medication Was Inappropriately Prescribed	Pharmacist Recommendation
ACEis (2)	Contraindications apparent (1)	Drug change: cease and initiate (1)
	Cautioning against toxicity (1)	Drug change: cease and initiate (1)
Diuretic (3)	Contraindications apparent (2)	Refer to prescriber (1) Dose decrease (1)
	Other drug selection problem (1)	Dose decrease (1)
Metformin (8)	Contraindications apparent (3)	Dose decrease (1) Drug change: cease (1) Drug change: cease and initiate (1)
	Prescribed dose too high (4)	Dose decrease (3)
	Toxicity caused by dose (1)	Drug change: cease (1)
ARBs (6)	Cautioning against toxicity (2)	Monitoring: laboratory test (2)
	Contraindications apparent (2)	Monitoring: laboratory test (2)
	Prescribed dose too high (1)	Dose decrease (1)
	Toxicity caused be dose (1)	Drug change: cease (1)
NSAIDs (1)	Cautioning against toxicity (1)	Monitoring laboratory test (1)
SGLT2 inhibitors (1)	Contraindications apparent (1)	Drug change: cease (1)

## Data Availability

The data presented in this study are available on request from the corresponding author. The data are not publicly available as restrictions apply to the availability of these data.

## Supplementary Materials

The following supporting information can be downloaded at https://www.mdpi.com/article/10.3390/medicina60050798/s1: Table S1: Modified DOCUMENT DRP and examples from HMR, Table S2: Modified DOCUMENT recommendations and examples from HMR.
