# Supplementary material for: Drug-Related Problems and Recommendations Made during Home Medicines Reviews for Sick Day Medication Management in Australia
Source: Medicina (Kaunas). 2024 May 11;60(5):798. doi: 10.3390/medicina60050798 (PMC11123247; doi:10.3390/medicina60050798)
Supplement: Supplementary file 1 [file medicina-60-00798-s001.zip › medicina-2947124-supplementary.pdf]

Table S1: Modified DOCUMENT DRP and examples from HMR.

Code	Drug related problem	Description	Example from HMR
D1	Duplication	Inappropriate use of two drugs from the same therapeutic class	"…given there is a duplication of vitamin D in the Caltrate supplement…"
D2	Drug interaction	Likely interaction between two prescribed drugs (no symptoms evident yet)	"Mr X is prescribed clomipramine, olanzapine and thioridazine and all of these can cause QT interval prolongation."
D3	Wrong Drug	Incorrect drug supplied, either by an incorrect doctor's prescription or incorrectly dispensed by the pharmacy	"Mrs X is administered carvedilol for the management of heart failure…carvedilol is a non-selective betablocker and can be associated with increased risk of bronchospasm noting that Mrs X does have a history of asthma…"
D4	Incorrect strength	Incorrect or no details about strength of medication supplied	N/A
D5	Inappropriate dosage form	Formulation is inappropriate in terms of the intended use of the product	N/A
D6	Contraindications apparent	Patient has a contraindication or precaution to the drug being used due to their medical conditions, or a	"She also has a history of glaucoma and recent ophthalmological visit highlighted increased

		previous allergy to the drug or drug group	intraocular pressure. Ongoing use of amitriptyline could further worsen glaucoma…”
D7	No indication apparent	No clear indication for the use of the drug	“She is taking the anticoagulant apixaban…although I could not determine an indication from the list of current conditions.”
D0	Other drug selection problem	Other drug selection issues, such as the patient is taking expired medication or a more effective drug than the one prescribed is available	“Nulax is a bulk-forming laxative and requires adequate hydration to be effective and given that Mr X is on the fluid restriction…they may not be effective.”
O1	Prescribed dose too high	Total daily dose exceeds guidelines, either due to reference dose ranges or patient parameters (age, renal function, etc.)	“If used for anticoagulation in the setting of AF, a lower dose of rivaroxaban at 15mg daily is recommended…”
O2	Prescribed dose too low	Total daily dose is not adequate for treatment	“She is only taking 480mg dose twice daily and her weight of around 58-59kg allows for dosing up to 1g per dose.”

O3	Incorrect/unclear dosing instructions	Specified dosing frequency/schedule or duration of treatment is unclear or incorrect	"It is an ocular steroid…it should be used under close supervision and PRN administration may not be optimal."
O0	Other dose problem	Other dose related problem, such as incorrect frequency or schedule	"Sometimes administration of atorvastatin can cause sleep disturbances…"
C1	Taking too little	Patient using too little of the medication due to forgetfulness or poor understanding of therapy	"Mrs X advised that she has been taking Symbicort Turbuhaler one daily. Currently it is prescribed as 1 bd."
C2	Taking too much	Patient using too much of the medication due to forgetfulness or poor understanding of therapy	N/A
C3	Erratic use of medication	Patient using medication on an erratic basis	"Mrs X has not been taking her Symbicort Turbuhaler, instead saying she only uses when necessary…"
C4	Intentional drug misuse (including OTC medications)	Suspected overuse of a drug that is potentially abused	N/A
C5	Difficulty using dosage form	Patient has a physical problem using the dosage form due to swallowing difficulties, manual dexterity, etc.	"Mrs X reported some difficulty swallowing large tablet size including fish oil as well as Panadol Osteo."

C0	Other compliance problem	Other compliance issues, such as patient choosing not to take the medication due to the product information or a media release, etc.	"There have been recent reports of difficulty getting warfarin packs due to communication issues between his current medical centre, pharmacy and group home…"
U1	Condition undertreated	Patient has a symptom or condition that is not currently being treated adequately	"He is administered mirtazapine 7.5mg nocte…reported his mood has been a bit low recently due to the recent death of his dog as well as being stressed…"
U2	Condition untreated	Patient has a symptom or condition that is not being treated	"Health summary documented his diagnosis of osteoporosis, but therapy was not listed, and I could not confirm if treatment is currently being given."
U3	Preventative therapy required	Patient requires additional therapy to prevent an adverse event occurring (due to patient's therapy, coexisting diseases or risk factors)	"The use of leuprorelin can be associated with low bone mineral density which increases the risk of osteoporosis...There could be benefit from the addition of a

			vitamin D and calcium supplement which may reduce the loss of bone mineral density.”
U0	Other untreated problem	Other untreated indication problem	N/A
M1	Laboratory monitoring	Patient requires a laboratory test, such as serum electrolyte or drug levels (no symptoms evident yet)	“It would be prudent for ongoing monitoring and review of thyroid function to ensure Mrs X remains in a euthyroid state”
M2	Non laboratory monitoring	Patient requires a non-laboratory test, such as BP, BSL or weight check (no symptoms evident yet)	“It would be prudent to monitor for any signs of postural drop for e.g. monitoring both sitting and standing blood pressure.”
M0	Other monitoring problem	Other monitoring problem, such as patient unable to afford monitoring	“Given that metformin usually does not cause hypoglycaemia, suggest withdrawal of BGL monitoring…”
E1	Patient requests drug information	Patient requests information about their medication	“Mrs also asked me about taking calcium tablets.”
E2	Patients requests disease management advice	Patient requests information about the management or prevention of a condition	N/A
E3	Confusion about therapy or condition	Patient has a poor understanding of their medical condition, but their medication compliance appears to be	“She may receive greater benefit from using her Symbicort

		adequate according to the dispensing history	regularly…however I am not sure she was convinced”
E4	Demonstration of device	Patient has a technical problem with the administration of a device	N/A
E0	Other education or information problem	Other education problem, such as another health professional requests information	N/A
N0	Clinical interventions that cannot be classified under any other category	Clinical interventions that the pharmacist feels does not belong elsewhere (must still be a clinical problem, not administrative)	“Mrs X did say that she was charted on mirtazapine ‘to improve her appetite’…not clear if Mrs X is also seen by a dietician.”
T1	Toxicity caused by dose	Patient has signs or symptoms of an adverse reaction that is likely to be dose-related	“She complained of daytime tiredness/drowsiness…I note pramipexole could contribute to feeling fatigue.”
T2	Toxicity caused by drug interaction	Patient has signs or symptoms of an adverse reaction that is likely to be related to the presence of an interacting drug	“There have been recent reports of vertigo… the combination of three agents in conjunction with his use of furosemide can increase the risk of postural hypotension.”
T3	Toxicity evident	Patient experiencing symptoms of toxicity where there is a suspected medication cause	“Mr X reported that he experienced symptoms of itchy skin…he is

			administered rivaroxaban which can be associated with pruritis…”
T4 **	Cautioning against toxicity	Patient at risk of toxicity/adverse drug reaction but no symptoms yet. Pharmacists may warn of side effects/cautions to take when using the medication e.g. risk of falls, sedation, etc.	“Prednisolone may reduce bone mineral density and Mrx X has a history of falls.”
T0	Other toxicity evident	Other toxicity suspected of being related to a drug	“I am not clear if her muscle discomfort is secondary due to statin therapy.”
NC **	Not clinical (NC)	Non clinical issues, often documentation/administrative discrepancies.	“Mrs X is allergic to Zocor, however, she has been receiving simvastatin 40mg at night time and as indicated as above she has been tolerating this medication.”

**New categories added to existing DOCUMENT system

Table S2: Modified DOCUMENT recommendations and examples from HMR.

Code	Recommendation	Description	Example from HMR
R1	Dose decrease	Pharmacist recommends the daily dose of medication is decreased	"…a gradual dose reduction of diazepam at night time could be considered."
R2	Dose increase	Pharmacist recommends the daily dose of medication is increased	"…perhaps addition of a lunchtime dose of quetiapine may be of benefit."
R3	Drug change	Pharmacist recommends a change in current medications, such as initiating or ceasing a medication	N/A
R3a **	Drug change: cease	Pharmacist specifies ceasing a medication	"…consider withdrawal of iron supplement if possible."
R3b **	Drug change: initiate	Pharmacist specifies initiating a medication	"Would he benefit from addition of vitamin D supplement for his osteoporosis?"
R3c **	Drug change: cease and initiate	Pharmacist specifies ceasing a medication and initiating another	"Suggest replacing Agarol with Movicol or lactulose"
R4	Drug formulation change	Pharmacist recommends a change in formulation that does not alter the drug or its total daily dose	"Omeprazole should not be crushed. Suggest replacing it with lansoprazole 15-30mg daily which can easily be dissolved in water."
R5	Drug brand change	Pharmacist recommends a change in the brand to	N/A

		improve compliance or due to stock unavailability, etc	
R6	Dose frequency/schedule change	Pharmacist suggests a change in the number of times per day or timing of the doses, without changing the total daily dose	"Suggest switching to mane dosing if ongoing poor sleep is a concern."
R7	Prescription not dispensed	Pharmacist does not dispense the prescription due to the circumstances, such as when the patient needs to visit the prescriber prior to dispensing	N/A
R8	Other changes to therapy	Pharmacist recommends another change to patient's current therapy	"Acnatac is likely to increase susceptibility to UV radiation, and so increased sun protection is recommended."
R8a **	Drug change: combination formulation	Pharmacist recommends to changing an individual drug formulation to a combined drug formulation.	"To reduce pill burden suggest using a combination product of calcium and vitamin D…"
R9	Refer to prescriber; prescriber to continue monitoring, review, etc	Pharmacist refers patient to their prescriber to resolve the DRP	"Given the history of smoking you may wish to consider monitoring for any underlying signs of COPD given the recent cough symptoms."
R9a **	Review prescribed medicine	Pharmacist recommends to specifically review the current prescribed medications	"In view of her weight gain reviewing the need for this supplement would be suggested."

R10	Refer to hospital	Pharmacist refers patient to the hospital to resolve the DRP	N/A
R11	Refer for medication review	Pharmacist recommends patient have a medication review to resolve the DRP (known as a Home Medications Review or HMR in Australia where a pharmacist visits the patient at home and sends a clinical review letter to their treating physician)	N/A
R12	Other referral required	Pharmacist refers patient to another health professional to resolve the DRP, such as a dentist, podiatrist, diabetes educator, etc	"Consider a referral to physiotherapy or exercise physiologist for a tailored exercise regime."
R13	Education/counselling session	Pharmacist provides a detailed counselling or education session to the patient to resolve the DRP	"We discussed her medications, what each one is used for."
R14	Written summary of medications	Pharmacist provides patient with a detailed list of their medications to resolve the DRP	N/A
R15	Commence dose administration aid	Pharmacist suggests that the patient start using a dose administration aid (such as a Webster pack or dosette box)	"It would be prudent to consider packing all of his medications for ease of administration and to reduce the risk of an inadvertent medication administration error."

R16	Other written information	Pharmacist provides other written information, such as Self Care cards	N/A
R16a **	Information to nursing staff	Pharmacist provides information to nursing staff to resolve DRP	"It would be prudent to ensure that staff are rinsing Mrs X's mouth after each use to reduce the risk of voice changes as well as oral thrush."
R17	Monitoring: laboratory test	Pharmacist suggests that the prescriber undertake some laboratory monitoring to monitor for DRP	"Given the use of carbamazepine consideration could also be given for assessment of LFT's noting the recent elevated GGT levels."
R18	Monitoring: non-laboratory test	Pharmacist suggests that the patient or prescriber undertake some non-laboratory monitoring to monitor for DRP	"…consider monitoring both sitting and standing blood pressures to help observe signs of a postural drop."
R19	No recommendation necessary	Pharmacist has investigated the problem and finds that the problem does not need to be addressed with any changes	N/A
R20 **	Nonclinical e.g. update document	Pharmacists makes a non-clinical recommendation to resolve issue.	"Consider discarding stock and to follow-up with pharmacy to ensure that new signing sheets do not continue to be sent due to risk of medication incident."

R0 **	Not classifiable	Pharmacist recommendation cannot be classified by any other category	"Is Ms X being treated for osteoporosis?"

**New categories added to existing DOCUMENT system
